# The Flow of Axonal Information Among Hippocampal Subregions: 1. Feed-Forward and Feedback Network Spatial Dynamics Underpinning Emergent Information Processing

**DOI:** 10.3389/fncir.2021.660837

**Published:** 2021-08-27

**Authors:** Yash S. Vakilna, William C. Tang, Bruce C. Wheeler, Gregory J. Brewer

**Affiliations:** ^1^Department of Biomedical Engineering, University of California, Irvine, Irvine, CA, United States; ^2^Department of Bioengineering, University of California, San Diego, San Diego, CA, United States; ^3^Center for Neuroscience of Learning and Memory, Memory Impairments and Neurological Disorders (MIND) Institute, University of California, Irvine, Irvine, CA, United States

**Keywords:** electrode array, entorhinal, dentate, CA3, CA1, axons, burst, power law

## Abstract

The tri-synaptic pathway in the mammalian hippocampus enables cognitive learning and memory. Despite decades of reports on anatomy and physiology, the functional architecture of the hippocampal network remains poorly understood in terms of the dynamics of axonal information transfer between subregions. Information inputs largely flow from the entorhinal cortex (EC) to the dentate gyrus (DG), and then are processed further in the CA3 and CA1 before returning to the EC. Here, we reconstructed elements of the rat hippocampus in a novel device over an electrode array that allowed for monitoring the directionality of individual axons between the subregions. The direction of spike propagation was determined by the transmission delay of the axons recorded between two electrodes in microfluidic tunnels. The majority of axons from the EC to the DG operated in the feed-forward direction, with other regions developing unexpectedly large proportions of feedback axons to balance excitation. Spike timing in axons between each region followed single exponential log-log distributions over two orders of magnitude from 0.01 to 1 s, indicating that conventional descriptors of mean firing rates are misleading assumptions. Most of the spiking occurred in bursts that required two exponentials to fit the distribution of inter-burst intervals. This suggested the presence of up-states and down-states in every region, with the least up-states in the DG to CA3 feed-forward axons and the CA3 subregion. The peaks of the log-normal distributions of intra-burst spike rates were similar in axons between regions with modes around 95 Hz distributed over an order of magnitude. Burst durations were also log-normally distributed around a peak of 88 ms over two orders of magnitude. Despite the diversity of these spike distributions, spike rates from individual axons were often linearly correlated to subregions. These linear relationships enabled the generation of structural connectivity graphs, not possible previously without the directional flow of axonal information. The rich axonal spike dynamics between subregions of the hippocampus reveal both constraints and broad emergent dynamics of hippocampal architecture. Knowledge of this network architecture may enable more efficient computational artificial intelligence (AI) networks, neuromorphic hardware, and stimulation and decoding from cognitive implants.

## Introduction

Routing of information through the hippocampal tri-synaptic circuit is widely viewed as the process that enables learning or remembering cognitive events as components of episodes. The inherent wiring architecture enables episodes to be completed from partial information or recognized for novel aspects or locations. We have considerable anatomical knowledge of the circuit, first described in 1911 by Ramon y Cajal as a sequential relay of connections among three anatomical regions within the hippocampus: from the entorhinal cortex (EC), to the dentate gyrus (DG) to the CA3 and CA1 (Andersen et al., 1971), before closing the loop back to the subiculum and entorhinal cortex. While this understanding is of a largely unidirectional circuit, except for the final feedback connection, control theory requires feedback in other subregions, as seen in major cortical areas.

### Motivation for a Reverse-Engineered Hippocampus

We know a lot about individual synaptic structures, synaptic machinery, transmitters and their receptors, voltage-gated channels, and action potentials, but less about how these components dynamically bind cell assemblies in each subregion and contribute to the whole flow of information and the specialization at each stage, leading to learning and remembering. In order to learn more about the spatial connectivity that emerges from connecting these subregions, here, we reverse-engineered the rat hippocampal formation by micro-dissecting these individual subregions from young postnatal animals and plated dispersed neurons into a novel four-compartment device. The four compartments were bridged by microfluidic tunnels that allow only axons to communicate between subregions. The entire system sits on top of a 120-electrode micro-array that monitors extracellular action potentials in the subregions and form pairs of electrodes in the tunnels to follow the flow of inter-regional action potentials and identify feedback routing.

One of the great wonders of experimental neuroscience is the emergent development of neural activity, modeled in culture, from isolated spherical cells extricated from the developing rodent brain, first as dendrites, then axons, and then synapses (Gross, 1979; Bartlett and Banker, 1984). While we know much about how the rich repertoire of spikes and bursts must coordinate the flow of information, the architecture for the coordinated flow is poorly understood. The intrinsic ability of isolated brain neurons to reconnect in an *in vivo* order has been demonstrated by a number of groups (Czarnecki et al., 2012; Downes et al., 2012; Kanagasabapathi et al., 2013; Dranias et al., 2013). Contrary to what might be expected in a uniform culture environment, we have found by reverse transcription polymerase chain reaction (RT-PCR) that subregions dissected from specific regions of the rat hippocampus (DG, CA3, CA1, and EC) maintain their subregion-specific gene expression (Brewer et al., 2013). Several laboratories have segregated two parts of the brain into a bidirectionally connected network (Kanagasabapathi et al., 2013; Bhattacharya et al., 2016; DeMarse et al., 2016; Poli et al., 2017, 2018a,b), cortical-striatal (Virlogeux et al., 2018). In a three-compartment culture of cortical-hippocampal-amygdala, Dauth et al. (2017) more rigorously showed unique patterns of protein expression by mass spectroscopy. This suggests that epigenetic switches were already set for the fate of the neurons to produce distinct expression profiles and, hence, behave in different electrophysiological capacities. In this study, we explicitly describe these electrophysiologic distinctions with a novel four-compartment device to enable the characterization of spike dynamics in the primary input into the four major subregions of the hippocampus, namely, EC, DG, CA3, and CA1 in a realistic closed loop architecture.

### Technological Advances Using Microelectrode Arrays

This utility of brain investigation using micro/multi electrode arrays (MEAs) follows 40 years of progress in the development and use of cutting-edge microfabrication technologies with *in vitro* brain preparations, notably pioneering studies by Gross (1979) and Pine (1980, 2006) with cultured neurons, and the team of the authors (Novak and Wheeler, 1989) using current source density to better reveal the propagation of epileptiform activity in hippocampal slices. The growth of the technology, industry, and applications is related in a history by Pine (2006). The technologies have been used for new insights in epilepsy, stroke, learning/memory (examples: Shimono et al., 2002; Stett et al., 2003; Colombi et al., 2013) and for long-term cultured slices (Egert et al., 1998). MEA technology continually outpaces the science as several groups (Hutzler et al., 2006; Hierlemann et al., 2011; Ferrea et al., 2012; Yuan et al., 2018)^1^ have demonstrated arrays with electrode counts increasing from tens to thousands of electrodes and feature dimensions reduced from tens of microns to a few microns from slices and dissociated cells, giving detailed images of signal propagation recordings with variations in the conduction velocity of individual axons (Bakkum et al., 2013). Examples of how advances in MEAs enabled a new understanding of neuronal networks abound. For example, mouse organotypic slices coupled to high-density MEAs revealed faster spike rates in cortical preparations than hippocampal (Timme et al., 2014). Advances in network architecture introduced small-world, rich club topologies from dissociated hippocampal cultures (Poli et al., 2015; Schroeter et al., 2015). The potential for use of MEA technology in understanding the dynamics of neural activity has been studied by a number of groups using dissociated neural cultures (e.g., Gross, 2011) to explore learning capabilities (Jimbo et al., 1999; Marom and Shahaf, 2002; Cadotte et al., 2008). Control of the geometry of plated neurons has been used to understand the development of neural activity (Segev et al., 2002; Wheeler and Brewer, 2010), or to understand how information propagation depends on geometry (examples from the group of the authors: Alagapan et al., 2016; DeMarse et al., 2016). Flickering fluorescence from somal calcium sensors coincided with MEA measures of bursts of spikes, indicating spike burst-mediated opening of calcium channels (Jimbo et al., 1993; Takayama et al., 2009). Calcium imaging of cell assemblies and sequences is currently performed *in vivo*. The self-wiring of these circuits was described by Segev and Ben-Jacob (1998).

### Integration of Microfluidics and Electrode Arrays

One other technological advance has been the introduction of microtunnels for isolating axons (Taylor et al., 2003, Taylor et al., 2005), coupled with several reports that tunnels plus electrodes enable the recording of axonal activity and conduction direction (e.g., the group, Dworak and Wheeler, 2009). Rewiring between brain slices (Berdichevsky et al., 2010; Kanagasabapathi et al., 2013) and isolated hippocampal neurons (Brewer et al., 2013) was made possible by microtunnels and wells with axons communicating from one anatomical region to the next and microelectrode signal sampling (Poli et al., 2017). Here, we continue the rich history of *in vitro* 2D modeling of the complexities of the 3D brain using a four-compartment device over a 120-electrode array to access axons of transmission and disambiguate feed-forward and feedback flow of information between different subregions of the hippocampus.

### Information Transmission Ascertained From Axonal Spike Propagation

The small 0.1–1 μm diameter of axons within the hippocampus makes quantitative electrophysiology difficult and confounds the understanding of the outputs of processing in each layer. Hence, few studies describe the output of the EC through perforant path axons into the DG. DG mossy fiber outputs into the CA3 and the Schaffer collateral coupling from CA3 to CA1 are seldom studied, because the bundle of these small caliber axons is inaccessible to individual patch clamp technology. Consequently, it has been difficult to discern from a subregion how signals and information are processed for transmission into the next region, even in hippocampal slices or *in vivo* recording with tetrode arrays. Here, we overcome these problems by isolating individual axons in microfluidic channels that span two electrodes. The two sites permit the determination of directional propagation of action potentials, as pioneered by Dworak and Wheeler (2009) and utilized in the laboratory of the authors in two-compartment systems (Bhattacharya et al., 2016; Poli et al., 2017, 2018a,b). However, these two-compartment systems are likely limited by the absence of a circuit loop that we describe here in a four-compartment system.

### Hippocampal Network Architecture

To better understand the contribution of network architecture and spiking dynamics to separate information processing in each subregion of the hippocampus, here, we employ a unique four-chamber device to answer the question “how do the spike dynamics and axonal communication differ between subregions to process the flow of information?” Preliminary attempts at cross-correlations of spike times in the system revealed only non-significant differences between the subregions. By segregating feed-forward from feedback axon signals, here we report that spike patterns in individual axons reveal a rich repertoire of bursts that could be connected to their source and targets. In the feed-forward direction, subregional neuronal somata connect to their transmission axons, which in turn connect to their target subregional somata. In the feedback direction, the subregional order is reversed. While measures from individual axons provide clear indications of directionality, whether generalizations about the architecture of each subregion can be inferred requires statistical analysis of a number of such hippocampal networks. Subregional statistical differences in the spike dynamics of this emergent activity could contribute to understanding the network architecture and information processing. Decades of studies on hippocampal slices crudely stimulated the perforant path of Schaffer collateral bundles of axons without knowledge of the number of axons activated. Not surprisingly, large fractions of target neurons were stimulated in the CA3 or CA1 to produce a population spike, but not spike dynamics of individual neurons. Only recently has a cluster of target responses been associated with the spatial extent of each terminal field of axons (Hendrickson et al., 2016), followed by appreciation of differences in topographic organization along the longitudinal and traverse axes of the CA3 subregion (Yu et al., 2020). Pairwise spike correlations between cortical neurons in close proximity capture most statistical properties of a single neuron and provide a measure of population activity (Helias et al., 2014; Dettner et al., 2016). However, these populations could be more rigorously defined by their subregional somata to target axon and axonal source to target neuron as enabled here.

### Logarithmic Distributions of Spike Dynamics Underpinning Specificity in Network Architecture

Brain activity measures often exhibit logarithmic dynamics (Buzsaki and Mizuseki, 2014) that turn multiplicative functions into addition and division into subtraction (Polsky et al., 2009; Silver, 2010). Knowledge of this network architecture may apply to neuromorphic engineering in which a key pursuit is to mimic the extreme efficiency of memory storage, processing, and retrieval of vast amount of information that is routinely presented to a relatively small region of the brain, the hippocampus. A better understanding of hippocampal spatial dynamics may enable more efficient computational AI networks and neuromorphic hardware, as well as suggest patterns for human brain stimulation and decoding from cognitive implants.

## Materials and Methods

### Neuronal Network Culture in a Four-Chamber Device

**FIGURE 1:**
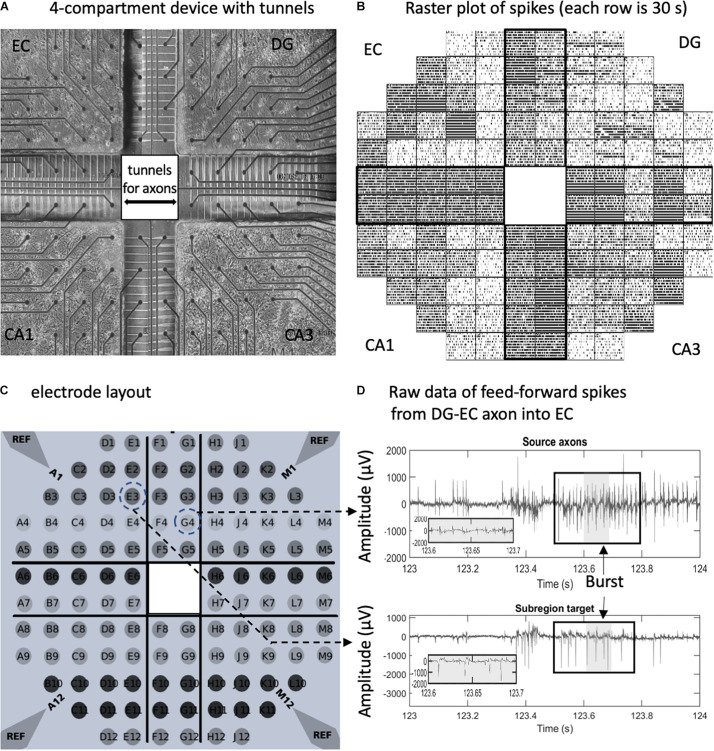
*In vitro* neuron culture of four separate hippocampal subregions with axonal interconnections aligned on a 120-electrode array. **(A)** Phase contrast image after 3 weeks in culture. Spacing between the 30-μm diameter electrodes is 0.2 mm. **(B)** Raster plot of spikes detected on individual electrodes over 5 min (30 s/row). Each tick is one spike. **(C)** Electrode array layout. Reference ground electrodes are seen in the corners. **(D)** Electrical feedback signals from the indicated electrodes with source axons in the dentate gyrus (DG)- entorhinal cortex (EC) tunnel appearing to elicit a response in the EC target. Large spike signal to noise ratios without high-pass filter. Box indicates a burst in spikes. Gray area is enlarged.

Glass multi-electrode arrays were substrates for the culture of neuronal networks (MEA120 with one hundred twenty 30-μm diameter electrodes spaced 0.2 mm apart, Multichannel Systems, Reutlingen, Germany). The substrates were cleaned according to instructions of the manufacturer. A custom polydimethylsiloxane (PDMS) device was aligned to the electrodes (Figure 1). Each of the four wells was 9.7 mm^2^ by 1 mm high. The wells were connected by 51 microfluidic tunnels 3-μm high × 10-μm wide × 400-μm long spaced 50 μm apart (Bhattacharya et al., 2016). The 120 electrodes allowed 19 electrodes in each subregion and 2 electrodes under each of 5 of the 51 tunnels between each subregion, thus monitoring about 10% of the axons between subregions. Details of the design and architecture of a two-compartment version were reported previously (Bhattacharya et al., 2016). The array with the attached device was activated with an air/oxygen plasma for 2 min to promote adhesion. The tunnels were filled with 70% ethanol to displace air. The ethanol was quickly replaced with poly-D-lysine (100 μg/ml, 135 kD, P6407; Sigma-Aldrich, St. Louis, MO, United States), which was allowed to attach overnight. Polylysine was aspirated and rinsed once with 18 M Ohm deionized water and allowed to dry. The hippocampal subregions were micro-dissected from postnatal day 4 Sprague–Dawley rat pups (Mattson et al., 1989; Brewer et al., 2013) under anesthesia as approved by the UC Irvine Institutional Animal Care and Use Committee. Brain cells were dissociated and plated at 1,000 cells/mm^2^ for DG (including the hilus), 330 for CA3, 410 for CA1, and 330 for EC (including subiculum). These densities were chosen to mimic the ratio of neuronal densities *in vivo:* EC-DG 1:3, DG-CA3 3:1, CA3-CA 11:1.25, and CA1-EC 1.25:1 (Braitenberg and Schuz, 2004). The plating and culture medium was NbActiv4 (BrainBits, Springfield, IL, United States) to promote synaptogenesis closer to *in vivo* densities (Brewer et al., 2008; Poli et al., 2018b). This medium is the classic Neurobasal/B27 medium (Brewer et al., 1993) supplemented with creatine, cholesterol, and low levels of estrogen. For comparisons with hippocampal slice physiology, this 270-mOsm culture medium contains (in mM) 66.3 NaCl, 26 NaHC0_3_, 5.36 KCl, 1.8 CaCl_2_, 0.81 MgCI_2_, 0.9 NaH_2_P0_4_, 0.2 Fe(N0_3_)_3_, 25 glucose, 0.23 pyruvate, 10 HEPES, 18 of the 20 common amino acids (minus excitotoxic glutamate and aspartate), four vitamins (biotin, vitamin E, vitamin E acetate, and selenium), albumin, three anti-oxidants (glutathione, superoxide dismutase, and catalase), two essential fatty acids (linoleic and linolenic acid), five hormones (T3, progesterone, insulin, corticosterone, and retinyl acetate) and several other ingredients (carnitine, ethanolamine, galactose, putrescine, and transferrin) optimized for hippocampal neuron survival *in vitro*. The cells in 10 μl of medium were plated into the wells sequentially. After 30 min in the incubator to allow for adhesion, the dish was filled with 1 ml of the medium that connected all the chambers. The cultures were capped with a Teflon sheet and incubated for 21–26 days in 5% CO_2_ and 9% O_2_. One-half of the medium was changed every 7 days. Activity was recorded after 20–25 days in culture, a period when cultured networks are reaching stability (Le Feber et al., 2009). Recordings were collected 2–5 days after medium change. In this study, final cell densities were not measured. Although glial densities were not measured, similar cultures contained 20% astroglia (Boehler et al., 2007).

### Multi-Electrode Array and Recording

Recordings were made on the 120-electrode microarray with a Multichannel Systems MEA120 1100× (Multichannel Systems, Reutlingen, Germany) amplifier. For this report, only spontaneous activity was analyzed with the MCRack software at a sampling rate of 25 kHz at 37°C for 5 min, in humidified 5% CO_2_, 9% O_2_, as in culture. Movement from the culture incubator to the amplifier often resulted in low levels of spiking. Recordings were initiated after several minutes, shortly after stable activity was seen in 80% of the tunnels. Arrays with less than 80% active tunnels or that had poor growth in one of the subcompartments were rejected for recording.

### Spike/Burst Detection and Dynamics

Spikes were detected after high-pass 400-Hz finite impulse response (FIR) filtering and then applying the Precision Time Spike Detection (PTSD) algorithm (Maccione et al., 2009) using the SpyCode MATLAB toolbox (Bologna et al., 2010). The differential threshold was chosen as nine times the standard deviation of the biological and thermal noise of the signal that was calculated for 200-ms contiguous windows. The PTSD algorithm searches for a peak near the threshold crossing for a time window as defined by peak lifetime period parameter, set to 1 ms. Additionally, the refractory period (dead time) was set to 1.6 ms to maximize spike counts and minimize complex spikes (Narula et al., 2017). Bursts were identified as four or more spikes with a maximum interspike interval (ISI) of 50 ms (Bhattacharya et al., 2016).

### Spike Sorting and Propagation in Axons

Sorting was not necessary for the subregions, because low cell density enabled the electrodes there to mostly detect signals from a single neuronal soma. Individual axons were detected in a tunnel by performing spike sorting on the two electrodes to detect single units (Supplementary Figure 1). Spike sorting was performed offline using the open-source MATLAB toolbox wave_clus (Quiroga et al., 2004). Single units were identified by performing feature extraction followed by unsupervised clustering. Haar wavelet transform with four-level multiresolution decomposition was used to reduce the dimensionality of the spike waveform. Unsupervised selection was used to identify 10 wavelet coefficients with the largest standard deviation between clusters of identified spike features. Superparamagnetic clustering was performed on the wavelet feature to identify single units. The temperature parameter of supraparamagnetic clustering (SPC) was varied from 0 to 0.2 in increments of 0.01, and the highest temperature with more than 60 spikes was chosen as the parameter for spike sorting. Additionally, clusters with refractory violations and temporal instability were rejected, as prescribed by Hill et al. (2011).

After spike sorting, a normalized matching index (NMI) was used to identify single units in the two electrodes that belong to the same axon (Poli et al., 2018b). This was done by comparing the timing of all the spikes of sorted units on one electrode to those on the second electrode in the same tunnel (Supplementary Figure 1). To associate units that belong to the same axon, an NMI algorithm is calculated for every combination of the units in each two-electrode tunnel, as shown in Supplementary Figure 1D. Paired spikes were identified as spikes with a delay between 0.1 and 1 ms, corresponding to the physiological propagation velocity of action potentials in an axon (0.2–2 mm/ms), adjusted for spike rate. NMI is used to quantify the temporal overlap between the two spike trains.

$$NMI=\frac{No.ofpairedspikes}{\text{max}\left(totalNo.ofspikesperspiketrain\right)}$$

The combinations of single units from the two electrodes above a threshold normalized matching index >0.2 were marked as belonging to the same axon. This NMI threshold was set to identify sufficient synchrony in spiking by systematic variation from 0.15 to 0.25. The value of 0.2 as a threshold was chosen to maximize the detection of axons while removing spurious matched units that showed low spiking synchrony and had no predominant direction of spike propagation from the histogram. Then, conduction time histogram is calculated for the matched units, as shown in Supplementary Figures 1F,G. The direction of spike propagation was identified using the peak of the histogram. The axons were said to have feed-forward propagation when the spike propagation occurred in the direction of the native flow of information in the tri-synaptic hippocampal loop.

### Probability Distributions

The distributions of spike dynamics that follow log-log distributions (interspike interval, spikes per burst, and inter-burst interval) were visualized as complementary cumulative probability distributions (CCDs) (Newman, 2005). The bins for the histogram were logarithmically spaced with the number of bins set to 50 per order of magnitude. The scaling parameter α is calculated by computing the slope of the linear regression line that fits the CCD (Arnold, 1983) after log transforming X and Y axes. The log transformed linear model was log10(P) = α^∗^log10(t) + c. The minimum and maximum time limits for the best fit (highest R^2^) were determined by initially setting the cumulative probability range between 0.1 and 1. This was followed by performing a grid search to find the local maximum for R^2^ when the time limits were varied by, at most, 50% using a step size of 5%. In the case of the inter-burst intervals without a clear single curve fit, a piecewise linear fit using the same method was applied twice, and the point of intersection was identified between the two linear fit lines. The initial minimum and maximum time limits for “up-states” (corresponding to the faster bursting) were set to the cumulative probability of 0.1 and 1. The minimum of the “up-states” was used as the maximum time limit for the “down-states” (corresponding to the slower bursting), and the initial minimum was set to the cumulative probability of 0.01. The burst dynamics measures were better fitted by a lognormal distribution, and probability distribution was analyzed using the histogram on a semi-log plot with logarithmically spaced bins, with the number of bins set to 100 per order of magnitude. The mode was calculated by fitting a normal distribution to the histogram and identifying the peak, and the standard deviation is calculated using the standard equation of lognormal distribution.^2^

$$Modeoflognormaldistribution={\text{e}}^{\left(m-\frac{s^{2}}{2}\right)}$$

The mean and SD, m and s, were estimated by fitting a normal distribution on log-transformed quantities.

### Network Graph Visualization

To correlate axonal activity with subregional source or target, average firing rates were computed for each 1 s of recording. Graphical connections were evaluated by linear regression between the pairwise combinations of each axonal and subregional firing rate. Slope values were interpreted as a measure of the strength of the connection, and R^2^ values were interpreted as a measure of reliability. In order to remove insignificant connections and for the sake of clarity, the linear models were analyzed when *R*^2^ > 0.2, and slope > 0.1.

### Data Processing and Statistics

Data were analyzed with custom MATLAB 2019b scripts (Supplementary Table 1). Statistical significance of the difference in slopes was evaluated in MATLAB by analysis of covariance (ANCOVA) with alpha set to 5%, followed by Tukey’s HSD (honestly significant difference) test. The significance of the difference in means was analyzed by analysis of variance (ANOVA), followed by Tukey’s HSD test. The null hypothesis was rejected for *p* < 0.05, adjusted for multiple comparisons. Significance tests are specified for each type of analysis within the figure legend. Data were analyzed for 10 separate networks (n). Scripts and spike time data are available upon request.

## Results

### A Reconstructed Hippocampal Network for Robustly Monitoring Axonal Communication

**FIGURE 2:**
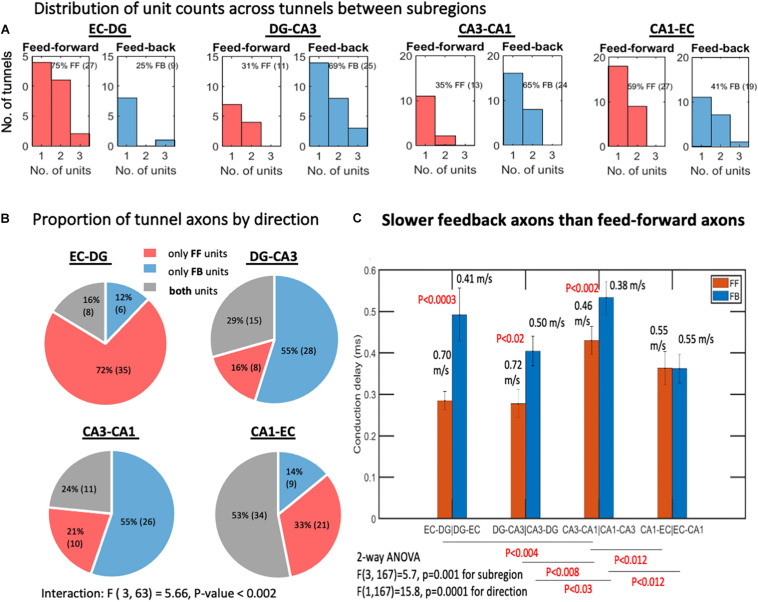
Feed-forward (FF) and feedback (FB) units between the subregions. **(A)** Interregional axons by subregions that they connect. Most tunnels were populated by a single axon. Two or three axon units were also detected. Spike sorting and subsequent determination of direction of propagation yield substantial numbers of feedback units in each subregion (*n* = 8 arrays). **(B)** EC-DG tunnels predominantly contain feed-forward axons, implying that the direction of information flow is predominantly feed-forward, whereas DG-CA3 and CA3-CA1 contain significantly more tunnels with feedback axons or bidirectional (containing both FF and FB units). **(C)** Slower feedback velocities of spike propagation than feed-forward axons by 2-way ANOVA with Tukey HSD subtests of the confidence interval overlap. *p*-values above bars are for the feed-forward vs. feedback comparison. Bars are mean and 95% confidence intervals of conduction delays. Corresponding conduction velocities are also indicated above bars for the 0.2-mm electrode spacing. *N* = 10 arrays.

In order to reveal the network architecture of communication between the four subregions of the hippocampal formation, we designed a four-compartment device with narrow 3 μm × 5 μm microfluidic tunnels that promote axonal but not dendritic entry (Figure 1A). These tunnels were previously shown to be axon-selective over dendrites (Taylor et al., 2003; Dworak and Wheeler, 2009) if their length was over 300 μm (Wang et al., 2012). Alignment over an MEA (Figure 1C) allows the recording of a rich repertoire of spontaneous signals and low noise (Figures 1B,D). Spontaneous spiking activity from 10 arrays was used for this study (Supplementary Figure 2). As previously demonstrated for axons communicating between two compartments of DG and CA3 (Narula et al., 2017), the tunnels mostly contain only one or two axons each (Figure 2A). Neurons isolated from distinct postnatal hippocampal subregions and grown *in vitro* maintain their distinct developmental subtypes of gene expression despite a common culture medium (Brewer et al., 2013). Although we can electrically monitor axons in five of the microtunnels in each of the bridges between compartments, the design incorporates 10 times that many microtunnels for maximum axonal communication between the subregions (DeMarse et al., 2016).

### Identification of Feed-Forward and Feedback Axonal Communication by Spike Sorting and Timing

Because of the high resistance in the 3 μm × 5 μm × 400 μm axon microfluidic tunnels, spike amplitudes are often in the mV range (Dworak and Wheeler, 2009), while out in the subregions only soma have sufficient current source density to signal a spike (Nam and Wheeler, 2011). In the microfluidic channel, there is room for many 0.1- to 1-μm diameter unmyelinated axons (Bartlett and Banker, 1984). To determine how many axons were in a tunnel and whether they were conducting spikes in the feed-forward or feedback direction, we first sorted spikes at each electrode by their waveforms, assuming each axon produced a unique waveform (Supplementary Figures 1A,B). Since each axon would be coupled differently to each of the two electrodes in the tunnel, waveforms on the two electrodes would differ and could not be used for the identification of a unique axon. To identify unique axons, we required the conduction velocity of each action potential in one axon to be constant over the 200-μm spacing between electrodes. At an average of around 0.5 mm/ms for an unmyelinated axon (Colombe and Ulinski, 1999; Kondo et al., 2004; Dworak and Wheeler, 2009), we looked for delay times and directions between ±0.1 and 1 ms (0.2–2 m/s). Supplementary Figure 1C shows waveforms on adjacent electrodes in a tunnel with a 0.6-ms delay in the feedback direction (in this case CA1 to CA3). Supplementary Figure 1D shows a matrix of NMI for all possible combinations of the pairing of sorted units on the two electrodes, with three sorted units per electrode. NMI quantifies the fraction of total spikes that are within physiologic delay times corresponding to conduction velocities of 0.2–2 m/s. The distributions of these particular spike time delays allow the identification of feed-forward or feedback directions with a Gaussian distribution of delay times (Supplementary Figures 1F,G). The 0.2-mm distance between adjacent electrodes divided by the median delay time is the conduction velocity (Supplementary Figure 1E). Sorted units that are identified as corresponding to the same axons are paired by choosing the combinations with maximum NMI, for all NMI >0.2. The conduction velocities were calculated for only the paired axons, and the others were shown blank here.

The trisynaptic circuit was first envisioned as a simple forward flow of information. Later, anatomy and physiology identified large numbers of inhibitory neurons within the hippocampal formation, which would be needed to prevent runaway excitation. To the knowledge of the authors, although rich local inhibition is well-known (Geiller et al., 2020), the extent of inhibitory axonal communication between subregions is less clear (Penttonen et al., 1997) and not compared between each subregion. Based on a single sorted waveform passing over two electrodes, Figure 2 shows that a large proportion of the microfluidic tunnels between regions contained a single axon conducting spikes in either the feed-forward or feedback direction, while some contained two or three identifiable units or both feed-forward and feedback axons. The proportion of feed-forward and feedback units might be expected to be balanced, but we found that the EC-DG axons were predominantly feed-forward, while DG-CA3 and CA3-CA1 units were predominantly feedback (Figure 2B). Two-way ANOVA for the four subregions and two directions was significant for their interaction, *F*(3, 63) = 5.66, *P* < 0.002, implying that the proportion of feed-forward to feedback unit counts varied between subregions, and that feed-forward axons failed to predominate over feedback axons [*F*(1, 63) = 0.58, *p* = 0.7]. We examined the conduction delay for all identified feed-forward and feedback axons, since a difference would affect how fast the feedback would occur. Figure 2C shows that feedback was slower among three of the four subregions, averaging 0.46 m/s compared with the feed-forward 0.61 m/s. Since conduction velocity is proportional to the diameter of the axon (Hursh, 1939), feedback axons are likely to be smaller in diameter than feed-forward axons. Of the DG-CA3 axons configured in the four-compartment loop, 69% showed feedback connectivity, which was much stronger than the 19% feedback in the previous two-compartment unlooped DG-CA3 design [*F*(1, 12) = 9, *P* = 0.01] (Bhattacharya et al., 2016). This result highlights the importance of network architecture. Together, this diversity of mechanisms allowed the overall flow of information within the circuit to be relatively balanced. Further analysis here will describe the distribution of firing rates by subregion and direction.

### Spike Dynamics—Interspike Intervals in Axons and Subregions Were Log-Log Distributed

We examined six measures of spike dynamics in the axons communicating between subregions, as well as the subregions themselves, all contained in one large network over a 120-electrode array (see Supplementary Figure 3 for definitions): (1) interspike intervals, (2) percent of total spikes in bursts, (3) spikes per burst, (4) inter-burst intervals (burst rates), (5) intra-burst spike rate, and (6) burst duration. None of these could be well-fitted with a Gaussian distribution model that characterized a simple biological mechanism with noise; they were better fitted by log-log or semi-log (lognormal) distributions.

**FIGURE 3:**
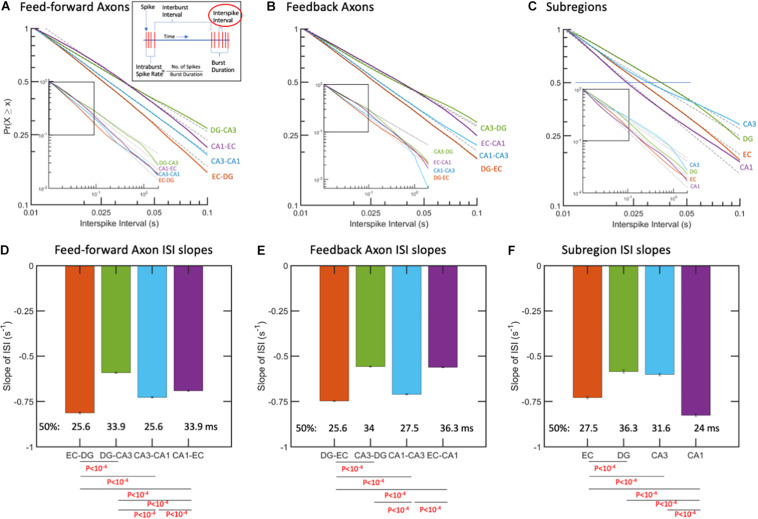
Interspike intervals (ISIs) followed log-log distributions (linear in log-log space). **(A)** ISIs as complementary cumulative probabilities in feed-forward axons. More spikes at shorter time intervals are from fastest spiking in EC-DG axons. Longer ISIs from slowest spiking axons are in DG-CA3. **(B)** ISIs in feedback axons. Fastest spiking in axons from DG back to EC. **(C)** Subregional ISIs are shortest (fastest) in the CA1 subregion. *R*^2^ > 0.99 for all fits. *N* = 10 arrays. **(D)** Feed-forward axon ISI slopes of which EC-DG is 27% faster than DG-CA3 and each of the others. The median ISI (*p* = 0.5) for EC-DG from A occurred earliest at 25.6 ms for EC-DG. **(E)** Feedback axon slopes of ISI distributions with DG back to EC significantly faster than the others and shortest median. **(F)** Subregion neuron ISI slopes with CA1 fastest. *P*-values calculated using ANCOVA followed by Tukey-HSD. The error bars represent the 95% confidence intervals obtained by ANCOVA.

Interspike intervals followed a log-log distribution from 0.01 to 1 s, corresponding to 1–100 Hz for all inter-regional axons (Figure 3). Thus, shorter times represent faster firing. This meant that the probability (P) of spike firing at a time, t, followed a linear model with slope m based on the equation P = t^*m*^. In log space, we have the linear equation Log(P) = m^∗^Log(t). All the data shown in Figure 3 for ISIs are well-fit by a single-power, one-characteristic slope, with R^2^ of 0.99 or better. The nature of this scale-free distribution (Beggs and Plenz, 2003; Newman, 2005) is that differing slopes would be most evident at long ISIs, but expansion to focus on the shorter times also would show this dispersion (Figure 3). Figure 3D shows that the slope of the curve fit for feed-forward axons from the EC to DG was significantly steeper by ANCOVA than between the other subregions, which indicated more events at shorter times and faster firing. Fifty percent of the EC-DG feed-forward axons (median) had an interspike interval less than 26 ms and were 27% faster than the slower DG-CA3 axons with longer spike intervals. From Figure 3E, it can be seen that the feedback ISIs are the fastest from the DG back to the EC. Figure 3F shows that the median ISI in the CA1 subregion is 24 ms and 41% faster than the slowest DG subregion spiking. Note that the log-log distribution cannot be accurately described by a certain mean and standard deviation. Median values were centered around 30 ms for feed-forward axons, 31 ms for feedback axons, and 29 ms for the different subregions, corresponding to 32–34 Hz firing rates, but this characteristic was not useful to discriminate between subregional axons or subregion wells because of the long-tailed distributions. This means that behaviors that are often characterized by mean spike rates are [Frame1]misleading and missing the full repertoire of a two-log range of spike rates. Log-log distributions enable a wider range of information transfer without a mean set point (Fagerholm et al., 2016). Data points longer than 0.05 s represent spike times between bursts, and some of the times shorter than 0.05 s are not in bursts, because they are not part of a sequence of four spikes with these short times.

### Spike Dynamics—The Majority of Spikes Were in Bursts

After examining all the individual spikes above, we examined their distribution in time. Figure 1D illustrates several clusters of spikes commonly called burst (Wong et al., 1979). We used a common definition for a burst as a group of at least four spikes with no more than 50 ms ISI (Brewer et al., 2013). Luczak et al. (2015) called these bursts packets of information, suggesting that they might be the main form of information transmission. Supplementary Figure 4 shows an average 60% of spikes in bursts for each of the axon directions and in the subregions. Spikes in bursts ranged from a low fraction of 0.52 in feed-forward axons from EC to DG to a high of 0.78 for feedback axons from DG to EC [ANOVA *F*(1, 15) = 3.3, *p* = 0.09]. By ANOVA, there were no significant differences in the fractions of spikes in bursts by subregions.

### Burst Dynamics—Spikes per Burst Were Log-Log Distributed From 5–100 Spikes

**FIGURE 4:**
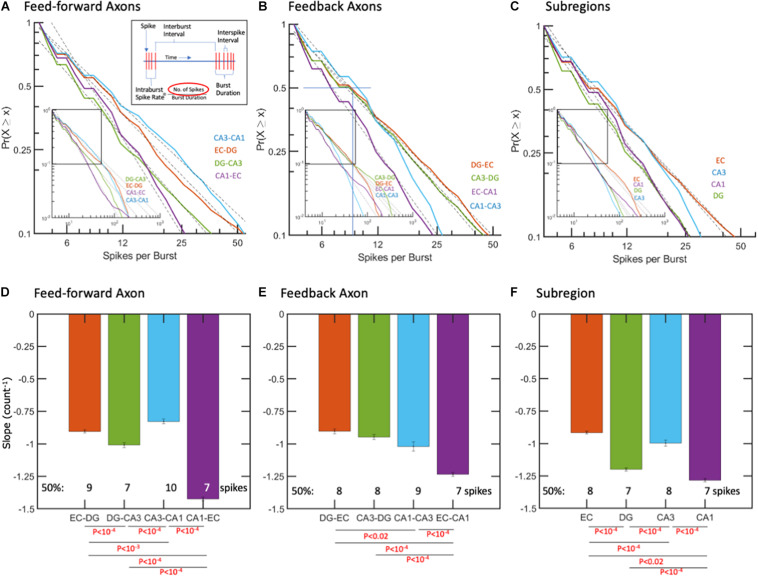
Spikes per burst follow log-log distributions. **(A,D)** Feed-forward axons in CA1-EC have the steepest negative slope with least spikes/burst. **(B,E)** Feedback axonal spikes per burst with steepest slope and least spikes per burst in EC-CA1 axons. **(C,F)** Subregional ISIs are shortest (fastest) in the CA1 subregion. *R*^2^ > 0.99 for all fits. The error bars represent the 95% confidence interval obtained by ANCOVA. Only the fraction of data between cumulative probability of 1 to 0.1 was used for fitting the linear model. *P*-values calculated by ANCOVA followed by Tukey HSD.

Spikes per burst might impact the strength of a packet of information and how long the activation signal was maintained, while other inputs were integrated. In contrast to the “burstiness” of cortical networks (Wagenaar et al., 2005, 2006; DeMarse et al., 2016), regional network-wide bursting was less common in these hippocampal subregions. As shown in Figure 4, spike counts per burst are well-fit by log-log models from 5 to about 100 spikes in both axon directions and in the subregions. Figure 4D shows feed-forward CA1-EC axons with the steepest negative slope and a median of 7 spikes per burst compared with the shallowest slopes in CA3-CA1 axons with 10 spikes per burst (ANOVA with Tukey HSD, *p* < 10^–4^). Differences in spikes per burst, as shown in Figures 4D–F, suggest subregional differences in coding for the flow of information.

### Inter-Burst Intervals Followed a Piecewise Log-Log Distribution, Suggesting Up-Down States

**FIGURE 5:**
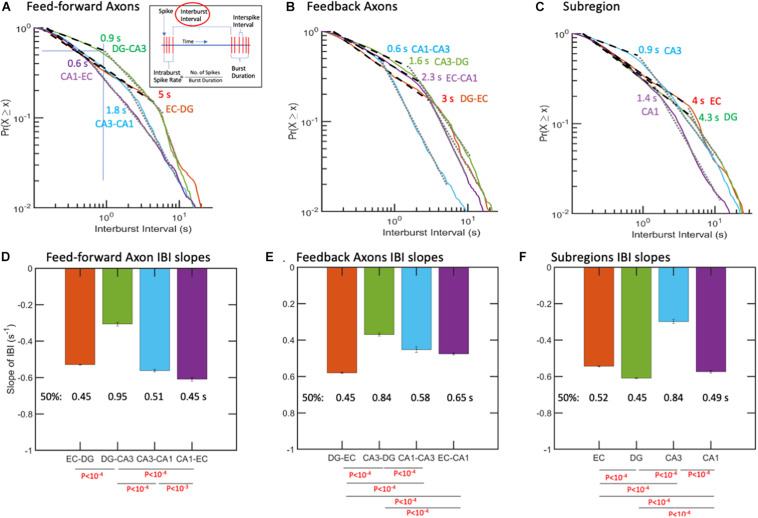
Inter-burst Intervals (IBIs) follow piecewise log-log distributions, suggesting up-down states. Points where log-log fits changed to a steeper slope are enumerated. **(A)** Feed-forward axons with least time of main state in DG-CA3 (most shallow slope), suggesting low range of up states compared with other axons there. Fifty percent of the up-states in DG-CA3 FF axons occur at times less than 1 s (horizontal and vertical lines). In contrast, 50% of the other feed-forward axons have IBIs of about 0.5 s. **(B)** Feedback axons in CA1-CA3 appear to switch from a domain of short inter-burst intervals to a longer domain around 0.6 s. **(C)** In the subregions, CA3 neurons switch from faster bursts to slower bursts around 0.9 s, while the transition in DG occurs only after 4.3 s. *R*^2^ > 0.99 for all fits. IBIs for **(D)** feed-forward axons, **(E)** feedback axons, and **(F)** subregion neurons. Least frequent bursting (longer IBIs) was in DG-CA3 feed-forward axons and CA3 subregion neurons. The error bars in the graphs represent the 95% confidence intervals obtained by ANCOVA. *P*-values calculated by ANCOVA followed by Tukey-HSD.

The time between bursts varied from 0.2 to 20 s with no central mean for feed-forward, feedback, or subregion activity (Figure 5). Distributions did not follow a single power, but in most cases were fitted nicely by two power functions. For feed-forward axons (Figure 5A), breakpoints ranged from 0.6 s for CA1-EC to 5 s for EC-DG. The shallowest slopes of inter-burst intervals were in the feed-forward DG-CA3 axons (Figures 5A,D), with the median occurring below the breakpoint of 0.9 s. For the shortest breakpoint at 0.6 s in CA1-EC axons, 40% of the bursts were spaced at times shorter than 0.45 s, but much more widely spaced from 0.6 to 20 s for the remaining 60%.

The short-spaced bursts could represent up-states, and the wide spacing between bursts could represent down-states (Li et al., 2009), likely controlled by inhibitory neuron activity (Zucca et al., 2017). Figures 5A,D show that these “up-states” for CA1-EC axons have more negative slopes (faster bursting) than shallower slopes of the DG-CA3 regional feed-forward axons. The CA1-EC axons transitioned from these high bursting rates to slower ones at only 0.6 s, while the EC-DG axons maintained higher rates of up to 5 s before breaking to slower rates. Conversely, half of the DG-CA3 axons transitioned from short inter-burst intervals to longer ones at 0.9 s. Only 50% of the feed-forward axons from CA1 to EC were in a faster-firing group (shorter interval). Over the next 1.5 log from 0.6 to 20 s, CA1-EC axons switched to a steeper slope, suggesting a switch from an up- to a down-state. The intercept for this switch in CA3-CA1 axons was at 1.8 s. In feedback axon bursting (Figures 5B,E), the breakpoints from faster burst rates (shorter times) to slower burst rates reached 0.6 s in CA1 back to CA3, and 3 s in DG back to EC, suggesting that feedback inhibition by burst rates lasted longer than the feed-forward excitation. In the subregions (Figures 5C,F), clear inflection points at shorter times (0.9 s) were seen for CA3 burst rates. The longest inflection times were observed for neurons in the EC (4 s) and neurons in the DG (4.3 s). Neurons in the CA1 were intermediate in their shift from a high to a low rate of bursting (1.4 s). Note that these are inter-burst intervals that translate to burst rates and not tonic intra-burst spike rates or phasic firing patterns from excitatory dominated rate controlled by gamma amino butyric acid (GABA) (Mann and Mody, 2010). Together, these data provided clear evidence of fast and slow burst rates for axons and neurons, suggesting up- and down-states of the flow of information through burst propagation.

### Intra-Burst Spike Rates Were Distributed Lognormally, Centered Near 95 Hz

**FIGURE 6:**
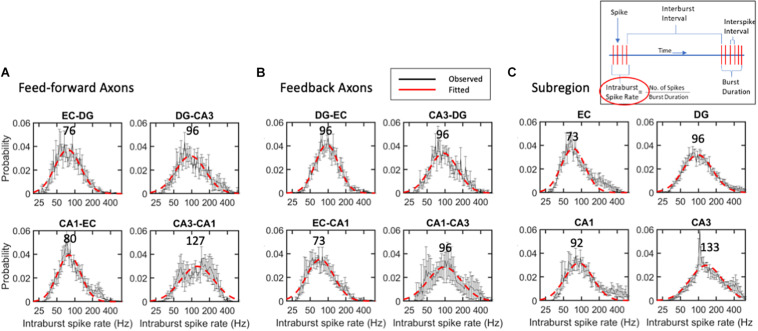
Intra-burst spike rates follow lognormal distributions without significant differences between **(A)** feed-forward axons, **(B)** feedback axons or **(C)** subregions. Error bars at each intra-burst bin are SE for *n* = 10 arrays. Red dashed line is best fit of a single Gaussian curve. Modes in Hz are indicated above curves. No differences were seen by ANOVA with Tukey HSD. All R^2^ of fits > 0.9.

**FIGURE 7:**
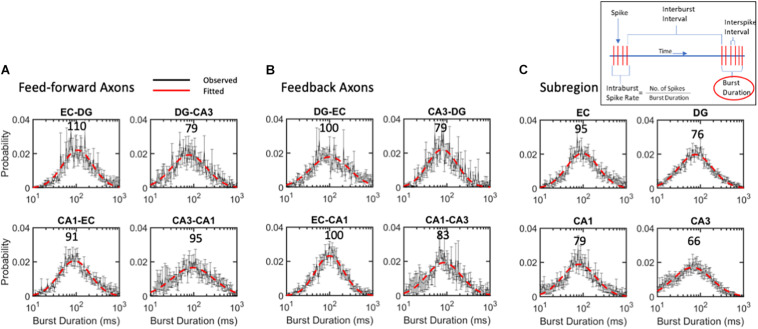
Burst duration varies over 2-order of magnitude with similar log-normal modes of 88 ms for **(A)** feed-forward axons, **(B)** feedback axons or **(C)** subregions. ANOVA with Tukey HSD indicated no significant differences between distributions. Error bars at each intra-burst bin are SE for *n* = 10 arrays. Red dashed line is best fit of a single Gaussian distribution. Modes in ms are indicated above curves. All R^2^ of fits > 0.9.

The strength of an axonal input and the number of synaptic vesicles releasing the transmitter will depend on the burst length and the spike rate within the burst. The criterion for a burst with at least four spikes, each arriving in less than 50 ms, was chosen to approximate the time constant for synaptic integration. These values put a lower limit on the spike rate of 20 Hz within bursts; the dead time of 1.6 ms puts a maximum rate approaching 500 Hz. Figure 6 shows the remarkably uniform lognormal distributions of spike rates. The distributions of these spike rates were noticeably long-tailed, better represented by a single logarithmic Gaussian transformation than a linear log-log model (e.g., R^2^ of 0.92 for the log-normal fit and 0.58 for a log-log fit of the same intra-burst spike rate data in the EC). From Gaussian lognormal fitting with R^2^ values greater than 0.9, we derived the mode or peaks of the distributions. None of these distributions were significantly different from the average burst rate of 95 Hz by ANOVA. This might be surprising in comparison with the significant differences in ISIs shown in Figure 3 but can be explained by three facts: (a) burst spiking is only about 60% of all spikes (Supplementary Figure 4); (b) a data subset has reduced power to reach significance; (c) all short times are not part of a burst. Burst firing rates in FF axons (Figure 6A) ranged from modes of 76 Hz for the slowest EC-DG axons to the fastest CA3 to CA1 axons at 127 Hz with an average feed-forward mode of 95 Hz. This order, which is different from the overall ISI, could reflect different portions of the distribution, and includes the longer burst durations of EC-DG shown in Figure 7. Feedback axon modes (Figure 6B) ranged from the slowest EC to CA1 axons at 73 Hz, to the others all at 96 Hz for an average feedback mode of 90 Hz. Subregional burst modes (Figure 6C) ranged from the slowest EC soma at 73 Hz to the fastest CA3 soma at 133 Hz and had an average mode of 95 Hz. Several higher rates that deviate from nearly perfect Gaussian fits were noticed as bumps in the distribution in feedback CA1-CA3 axons around 300 Hz (Figure 6B) and neurons in the EC (200 Hz) and CA1 and CA3 subregions (400 Hz) (Figure 6C).

### Burst Durations Were Distributed Lognormally Close to the Same 88-ms Mode

While the mode of intra-burst spike rates was 95 Hz, the burst duration could vary greatly, changing the drive into the target synapses. The burst duration of all communicating axons follows a lognormal distribution, with a mode centered around 88 ms (Figure 7). Burst length was lognormally distributed symmetrically from about 10 to 1,000 ms. Bursts in feed-forward axons ranged from modes of 79 to 110 ms with an average mode of 94 ms (Figure 7A). Feedback axon modes ranged from 79 to 100 ms with an average mode of 91 ms (Figure 7B). Subregional burst lengths ranged from 66 to 95 ms modes with an average mode of 79 ms (Figure 7C). None of these were significantly different from the overall burst duration mode of 88 ms by ANOVA of log-transformed Gaussian distributions.

### Correlations of Input Neuron Spike Rates, Axonal Transmission Rates, and Target Neuron Rates Enabled the Construction of Network Graph Maps of Connection Strength

**FIGURE 8:**
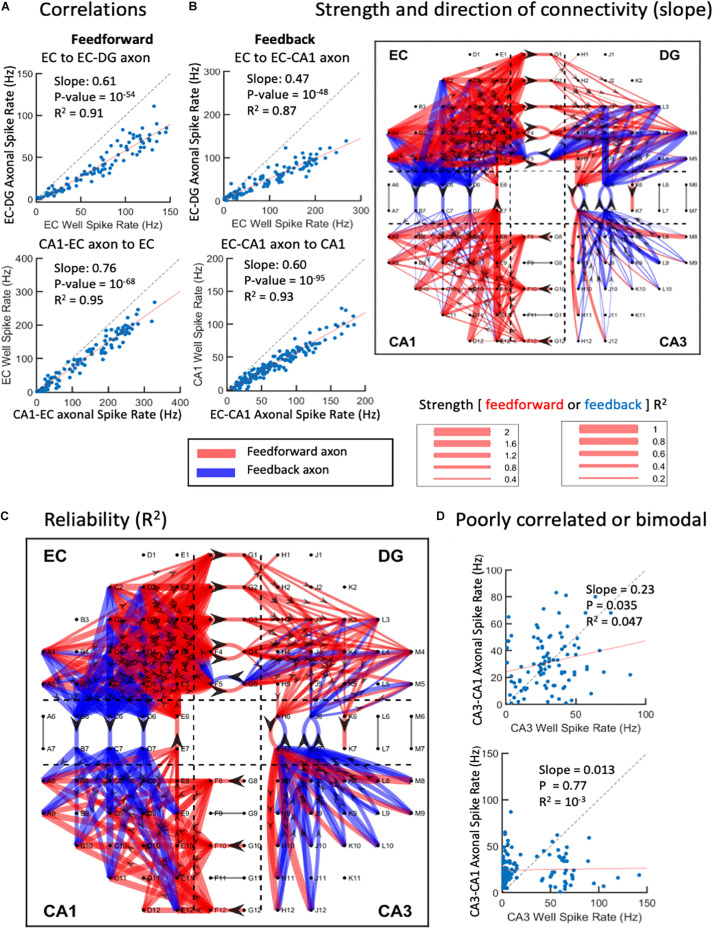
Graphical connections for one-network microelectrode array (MEA). **(A)** Spike rate correlations between source wells feed-forward to axons in tunnels or feedback from wells to axons. Each point is the spike rate for a 1-s window over the 300-s recording. Top two examples show strong source to axon correlations below unity (dashed line). Bottom two examples show axon to target correlations. **(B)** Magnitudes of slopes > 0.2 and *R*^2^ > 0.1. Feed-forward is in red, feedback in blue. Directionality indicated by arrowheads in axons. **(C)** Reliability of connections as correlation coefficients (R^2^). **(D)** Examples of poor correlations of CA3 neuron sources to CA3-CA1 axons. Bimodal distribution of CA3 activity in lower example (array 5).

The complexity of the above network dynamics might suggest difficulty in constructing the structural and functional connectivity of this *in vitro* network (Poli et al., 2015). Nevertheless, we examined the correlation of rates of each neighboring axonal tunnel to spike rates of each subregional source or target electrode, most commonly the activity of single neuronal soma. Since the directionality of the axonal transmission is determined, we are able to separate feed-forward correlations from feedback correlations, as shown in Figure 8. As each region was expected to process the incoming information differently, we did not expect subregional soma spike rates to elicit similar axonal spike rates, i.e., most rates would not be correlated with slopes of one. A rare case of recording the axon emanating from a subregional neuron electrode was unlikely, since each subregion electrode recorded less than 3% of the neurons in the region, and each microchannel with electrodes was only 1 of 51 microchannels (Figure 1A). Using the spike rate correlations, we measured axonal transmission strength as slopes, and reliability as R^2^ of the correlated source neurons through multiple routes. Figure 8A shows two feed-forward and two feedback examples with strong correlations. We noticed that source spike rates often led to spike rates in their targets below one. This could reflect low synaptic weights and the need for more than one axon to fire a target neuron or several sources of neurons needed to sum into the target axon. The segregation of the directions of the spikes of the axons in the tunnels allows the formation of a map of connectivity among all the subregions (Figure 8B) and their reliability (Figure 8C). It was clear that both feed-forward and feedback signaling occurred throughout the network. In agreement with Figure 2, feed-forward connections (red) were strongest from EC to DG, while feedback connections (blue) were enhanced from CA3 to DG. From CA3 to CA1, we could not find reliable firing rate correlations as exemplified in Figure 8D. Figure 1B and Supplementary Figure 5 show that the lack of linear CA3-CA1 connectivity was not due to lack of activity in CA3; CA3 neurons were spiking at high rates uncorrelated to spiking in axons leaving CA3 into the CA1 region. Baseline drift was not the cause, since recordings in different regions were acquired simultaneously and centered for DC baseline.

## Discussion

### Novel Device Enabled Measurements of the Directionality and Velocity of Axonal Communication Between Subregions of the Hippocampus

With a novel four-compartment device, we enabled the reconstruction of a hippocampal network comprising the entorhinal cortex, dentate gyrus, CA3, and CA1 subregions in a loop with axonal connections isolated in microfluidic tunnels. Electrodes in the subregions and two electrodes in each tunnel were designed to monitor the emergent activity of the direction of axonal propagation of action potentials. Most of the 3-μm-high tunnels contained only one or two axons. The directionality of axonal transmission enabled source, transmission, and target relationships. In the feed-forward direction, some subregional neuronal somata were connected to transmission axons, which in turn were connected to target subregional somata. In the feedback direction, the subregional order was reversed.

Directionality was surprisingly non-uniform with feed-forward axons from the entorhinal cortex to dentate gyrus predominating, while feedback axons prevailed from CA3 to dentate gyrus and CA1 to CA3. Reciprocal connections of the EC with CA1 have been reported (Rockland and Van Hoesen, 1999), as well as CA3-dentate (Penttonen et al., 1997; Lisman, 1999), possibly as axon collaterals of CA3 pyramidal neurons returning to innervate mossy fibers, hilar interneurons, and granule cells (Ishizuka et al., 1990; Li et al., 1994; Scharfman, 1994; Kneisler and Dingledine, 1995). However, CA1 to CA3 reciprocal connections have been incidentally reported from slice stimulation in CA1 (Andersen et al., 2000), although they are generally claimed not to exist (Schultz and Engelhardt, 2014), possibly because they have not been examined with modern tracer techniques.

In general, feed-forward axons exhibited faster spontaneous conduction velocities (0.5–0.7 m/s) than feedback axons (0.4–0.5 m/s). Based on cable models, velocity is proportional to axon diameter (Hursh, 1939; Hodgkin, 1954), suggesting that feedback axons were thinner, and that feed-forward axons were thicker for faster conduction velocity. To the knowledge of the authors, this distinction has not been reported, but should be confirmed at higher magnification by electron microscopy. These values were supported in general by the range of axon conduction velocities of 0.2–1.4 m/s seen in stimulated cortical neurons over an 11,000-electrode array *in vitro* (Bakkum et al., 2013). However, in contrast to the measurements over 0.2 mm, Bakkum et al. (2013) measured variations in conduction velocities of over 2.5 mm of axon length. In one axon, velocity was about 1.3 m/s in the first millimeter, then 1 m/s in the next millimeter, before tapering below 0.3 m/s, 2–2.5 mm from the soma. Another axon was fairly constant at 0.5 m/s, 0.2–0.5 mm from the soma. They did not distinguish between excitatory and inhibitory axons. More recently, conduction velocities of primary rat cortical axons in culture on a 19,584-electrode CMOS array were 0.48 ± 0.09 SD m/s (*n* = 1,086 neurons) (Yuan et al., 2020). Axons in mouse organotypic hippocampal slices increased action potential velocities with increased fiber diameter, measured by super-resolution stimulated emission depletion (STED) imaging (Chereau et al., 2017). In cortical neurons *in vitro* in MEA micro-fluidic channels such as those used in this study, Dworak and Wheeler (2009) found conduction velocities ranging from 0.2 to 0.8 m/s. In intact rat cortex *ex vivo*, De Col et al. (2008) found conduction velocities in unmyelinated axons activating the cranial meninges to depend on prior spike rate and sodium channel inactivation, ranging from 0.1 to 1.4 m/s, with a mean at 0.55 m/s. Measures of peripheral myelinated cat nerves of Hursh (1939) were much faster, ranging from 10 to 100 m/s. Rushton (1951) and, later, Waxman and Bennett (1972) compared these myelinated measures to those of slower unmyelinated cat C fibers from 0.2 to 1 μm in diameter and velocities from 0.8 to 2 m/s. The axon conduction speeds we report (0.2–2 m/s but more commonly ∼0.5 m/s) are generally consistent with measures in hippocampal slices (0.25 m/s, Schaffer collaterals; Andersen et al., 2000); 0.24 m/s, dentate granule cells (Schmidt-Hieber et al., 2007; Kress et al., 2008), and 0.38 m/s in CA3 (Meeks and Mennerick, 2007) or 0.22 m/s from CA3 to CA3 (Andersen et al., 2000). Reports from cultured neurons showed similar ranges (0.18–1.14 m/s) in a hippocampal study in the lab (Dworak and Wheeler, 2009; 0.4–1.4, peak at 0.6 m/s), the lab 0.6–1.4 m/s (Pan et al., 2011, 2014), ∼0.55 m/s, in hippocampus (Habibey et al., 2017) and 0.59 m/s (Tovar et al., 2018). Overall, the measured feed-forward spike velocities of 0.5–0.7 m/s are in the range of other *in vitro* and *in vivo* observations of unmyelinated axons, and we observe for the first time a slower velocity for feedback axons of 0.4–0.5 m/s. These results suggest that feedback axons are smaller in diameter than feed-forward axons, and that their slower speed may allow time for the propagation of multiple feed-forward spikes before the beginning of feedback inhibition.

### Log-Dynamic Hippocampal Networks

A second notable aspect of this article was the logarithmic relationships of spike and burst dynamics. Buzsaki and Mizuseki (2014) highlighted this feature of the brain, which is likely due to the multiplicative combination of a large number of variables. They provided examples of the lognormal power of brain waves, the proportion of cells firing in a 100-ms window (population synchrony), spontaneous firing rates in rat, monkey and human cortex, synaptic weights, spine size, and axon diameter. The stimulus amplitude of light and sound stimuli to the brain are detected over multiple orders of magnitude according to the Weber-Fechner law, logarithmic relationships. However, most research reports characterize the response to a stimulus, location, head direction, or even spontaneous activity in terms of mean firing rates (Coletta et al., 2018). Means are appropriate for Gaussian or normal distributions, but brain activity is mostly long-tailed with many events beyond the peak of activity. Because firing rate measurements rely on the time bin chosen as a denominator, we chose to quantify spontaneous spiking activity in terms of every ISI, independent of the time bin, except in the large limit of some bins. Long-tailed ISI distributions were observed early in cortical neurons on electrode arrays (Beggs and Plenz, 2003; Pasquale et al., 2008), mostly in terms of avalanches of connected activity with log-log, power-law distributions of the number of electrodes involved. The classical scale-free function requires that the distribution be independent of the size of the measurement bin. The approach of log bins contributed to the detection of scale invariance, as have been highlighted previously (McManus et al., 1987). This study is the first to rigorously compare subregional differences in the distribution of spike times, and is certainly unique in recognizing the log-log distribution of axonal spike times among the subregions. A plethora of non-linear mechanisms contribute to the log-log distributions of firing rates: multiple channel open times, channel densities, spine sizes, synaptic weights, receptor desensitization, inhibitory inputs, axonal and dendritic branching, and network cell assemblies. Compared with Gaussian distributions, functional advantages of log-log distributions include a wider range of coding, no central mean, and the ability to change multiplicative inputs into addition and transform division operations of inhibitory inputs into subtraction (Polsky et al., 2009; Silver, 2010). Cortical and subregional hippocampal networks reported here differ from other synchronized or wave-like oscillatory networks by operating at a log-log critical state that maximizes information transmission and fault tolerance while maintaining stability (Beggs and Plenz, 2003; Klaus et al., 2011; Dahmen et al., 2019). When normalized for all events, a single parameter of the slope of the cumulative distribution summarizes the spike distributions over two orders of magnitude from 10 to 1,000 ms. Distributions of spikes per burst and inter-burst intervals were well-fitted over a single order of magnitude. These spike-rate and spike-timing-based codes are part of a single continuum of coding in the hippocampus (Luczak et al., 2015; DeMarse et al., 2016).

#### Log Dynamic EC

Information processing in and between subregions of the hippocampal network was distinguished by the single time parameter of slope in the log-log distributions of interspike intervals, spikes per burst, and inter-burst intervals (burst rates). Here, we discuss each region in order around the network loop. Spiking within the EC region was faster than that in the neighboring DG, but with fewer spikes per burst and longer inter-burst intervals than in DG. Axons emanating from the EC and feeding forward into the DG region (EC-DG) exhibited the fastest spike rates (most short interspike intervals) and a high fraction of short inter-burst intervals. Of the feedback axons, DG-EC were also faster than most of the other feedback axons with shorter inter-burst intervals. Other *in vitro* studies on cortical rat neurons saw a wide range of bursting behavior with a similar log-normal median near 100 spikes/burst and spanning a range from 20 to 2,000 (Wagenaar et al., 2005). Power law distributions of ISIs are expected from a network of non-linear neurons (Persi et al., 2004). A more generalized Levy distribution incorporates break points in several log-log slopes of cortical neuron spike burst distributions as we observed. Spike rates within bursts were not measured, nor were comparisons made to hippocampal neurons or their axons. Rat *in vivo* measurements showed maximum spike rates in the EC, averaging 50 Hz (Barnes et al., 1990).

#### Log Dynamic DG

Within the dentate gyrus subregion, spike rates were lower than those within most of the other regions with fewer spikes per burst. Spike rates of output axons from DG into CA3 were the lowest feed-forward group. Spike rates of feedback axons from CA3 into DG were low as well. Feedback axons from the CA3 into the DG mossy cells have been documented *in vivo* (Penttonen et al., 1997) as well as axon collaterals back into the DG from the Schaeffer collaterals (Hendrickson et al., 2016).

#### Log Dynamic CA3

Within the CA3 subregion, spike rates were lower than those within most of the other regions reflected in the longest inter-burst intervals and shortest burst durations, even with high spike rates in these short bursts. These bursts drove short-durations of high spike rates of output axons from CA3 into CA1 accompanied by the highest proportion of spikes in bursts and shorter inter-burst intervals. Spike rates of feedback axons from CA1 into CA3 were high. At any point in time, the spike rate in the same neuron is modulated by the relative excitatory and inhibitory drive. As we saw poor correlations for CA3 with CA3-CA1 axons, Renart et al. (2010) more often observed the absence of correlations in somatosensory cortex in times of rat activity than inactive times. Their modeling showed that this could occur when recurrent inhibition was well-balanced by excitatory drive. Dahmen et al. (2019) also showed the range of such correlations centered at zero correlation in Macaque motor cortex. They noted that correlations near zero are characteristic of collateral inhibition-dominated networks, as we have seen in CA3 and would be expected from a network poised in the critical state (Beggs and Plenz, 2003; Dahmen et al., 2019).

#### Log Dynamic CA1

The twofold higher literature references to CA1 than CA3 or DG (Pubmed Search, January 2021; Komendantov et al., 2019) could arise from higher response rates to axonal stimulation. Among the four subregions, we saw the highest spike rates in CA1 with shorter inter-burst intervals (steepest slope in ISI distribution). Feedback of EC into CA1 was weaker. To complete the loop, CA1 output into EC was strong in overall spike rates and burst rates.

With these distinctions, it was surprising that intra-burst spike rates and burst durations followed lognormal distributions with similar mean rates and times distributed over two orders of magnitude. These signatures suggest that compared with overall spike and burst timing, intra-burst dynamics were regulated by a single, less complex but still non-linear mechanism. In contrast, variations in spike conduction delays (velocities) exhibited simpler (linear) Gaussian distributions, possibly due to thermal and measurement noise.

### Inter-Burst Intervals, Up-States, and Down-States

Despite the lognormal distributions of spike rates within bursts and burst durations, the probabilities of times between bursts followed log-log linear models. Luczak et al. (2015) called these bursts packets of information, suggesting that they might be the main form of information transmission in the behaving brain. With over 90% of bursts needing two slopes to fit a linear log-log model, inter-burst intervals appeared to be grouped into one of two characteristic probability distributions, one of shorter times between bursts and another of longer times between bursts. The shorter time group was consistent with commonly observed up-states and longer time intervals with down-states (Battaglia et al., 2004; Gretenkord et al., 2017; Tatsuno et al., 2020). Note that the measures are longer times between bursts, not a transition from bursts to non-burst spikes. These observations make common methods of average spike rate measures untenable if the distributions are log-log, with no central mean. Indeed, we found the CA3 subregion to stand out with a particularly short duration between bursts, breaking to longer duration down-states after only 0.9 s, in contrast to breaks of 4 s or longer for the up-states of EC and DG. Information processing within these regions results in feed-forward axonal transmission at high burst up-states of up to 0.9 s in DG to CA3, while up-states last up to 5 s in the EC-DG axons. Feedback axons from the DG to EC have the steepest slope with characteristic up-states extending out to 3 s. A mechanism for up- and down-states involves periodic variations in membrane potential, depolarizing toward and hyperpolarizing away from threshold (Steriade et al., 1993), varying at somewhat periodic 0.5–3 Hz. The relationship of 1–3 Hz delta slow waves to the slower burst rates in CA3, DG-CA3, and CA3-DG will be the subject of a third article in this series on slow waves in axons.

### Network Graph Maps Differences in Information Processing

The reconstructed hippocampus displayed connectional diversity between the subregions. Strong feed-forward axons emanated from the EC toward the DG, while strong feedback axons were directed from the CA3 into the DG. The majority of source to axon and axon to target relationships were linear. This result was surprising given the non-linear, logarithmic spike and burst dynamics reported in the rest of the article. Logarithmic relationships can arise from the integration of several linear functions (Newman, 2005). By calculating firing rates at 1 s, we could be measuring mostly single bursts or packets of information. In these bursts, the spike rate of a single axon is reliably transmitted (*R*^2^ > 0.1) from the source *via* an intermediary neuron to an efferent axon or from an interregional axon to the target. These relationships are characterized by a variety of slopes. Slopes above 1 suggest frequent co-incident axon spikes that undergo spatiotemporal integration to excite a target neuron, as in about one-third of the outputs from EC toward DG. A preponderance of slopes centered close to unity connected the EC forward into axons targeting the DG. This suggested reliable excitation unbalanced by inter-regional inhibition until later stages. All the other subregional connections ranged between slopes of 0.1 and 2. Significant, reliable slopes could arise from a number of factors. Low slopes could be caused by the local inhibition of excitation and/or a target neuron with weak synapses that need excitation from multiple axons to fire and/or temporal and spatial dendritic integration and sparse coding (Poli et al., 2017). Linearly unreliable connections (*R*^2^ < 0.1) with slopes below 0.1 might suggest a plethora of weak synapses or targets that require large numbers of synchronous inputs to achieve target neuron spiking (Poli et al., 2018a; Sherrill et al., 2020). Such weak pairwise correlations that we found here that differed between subregions provide the underpinning of emergent spatiotemporal patterns of population activity, as highlighted by Yu et al. (2020) in earlier studies by Halliday (2000); Schneidman et al. (2006), Kriener et al. (2009), and Renart et al. (2010). Earlier studies also found evidence for sharing propagation of the correlation through multiple layers (Kumar et al., 2010; Rosenbaum and Josic, 2011; Rosenbaum et al., 2017; Darshan et al., 2018). During monocular deprivation, network point to point spiking correlations change in the rat visual cortex, suggesting the importance of this regulation for maintaining homeostatic balance in the network. Further details on edges, hubs, and temporal dynamics, such as axonal synchrony, will be the focus of a second article in this series.

### Limitations

Disadvantages of the system include a 2D simplification of the 3D brain and the lack of lamellar organization of the mammalian hippocampus. Also limited are the thousands of neurons in each subregion compared with numbers approaching a million in the rat hippocampus (Braitenberg and Schuz, 2004). This likely limited the diversity of activity that we observed. The network lacked thalamic inputs or subcortical modulatory inputs, perhaps also limiting the repertoire of *in vitro* responses. The four subregions included combinations of further divisions such as the hilus with the DG, CA2 with CA3, and subiculum with EC. The device design here omitted EC to CA3 feed-forward activation through Schaffer collaterals. The study in progress incorporates micro-fluidic channels for this pathway. Further pharmacologic inhibition of GABA receptors is needed to establish that feedback axons are inhibitory. The attempts at waveform classification or spike rates to identify inhibitory axons proved unreliable, as others have found (Becchetti et al., 2012; Rogers et al., 2021). In an earlier two-subregion device, we found about 47% GAD67 reporter-tagged neurons in DG (inhibitory/total cells) opposing CA3, and only 10% GAD67 neurons in CA3 (Brewer et al., 2013); however, these could be mostly local inhibitory neurons. This report also showed GAD65 immunoreactive axons in microfluidic channels, supporting at least some inhibitory directionality seen here by the directionality of conduction velocities. Here, we have focused on differences in spatial dynamics between the subregions that include numerous connections over a fixed time of 5 min. A finer detail is expected in the next article in this series from analysis of the temporal dynamics to determine how many of the pathways exemplified are active during each burst of activity. In a third article, we also intend to integrate information on the slow waves from 1 to 300 Hz that we observed but were filtered out in this study. In a fourth article, we will delineate how this architecture can be used to learn patterns of stimuli, distinguish small differences, and complete partial inputs of prior patterns.

### Application

The approach of reverse engineering a hippocampal network fills the meso-scale gap in knowledge between micro studies on synaptic inputs to single neurons and millimeter-scale macro studies on functional magnetic resonance imaging. The patterns of feed-forward and feedback dynamics among different subregions of the hippocampus may hold the key to memory processing and consolidation for brain circuit prosthesis (Song et al., 2018). The feedback pathways are often related to the control of forwarding neuronal transmission, particularly in modulating excessive excitatory stimulations downstream (Yu et al., 2020). Repeating stimulation patterns, once learned, may elicit feedback transmission to decrease further stimulation of the same patterns to preserve neural subnetwork bandwidth and, thus, improve efficiency (Bein et al., 2020). Multiple hierarchies of feed-forward-feedback interactions may be the key to exponentially increase neuronal efficiency. This mode of memory formation and consolidation is consistent with how other parts of the brain function, such as the way the tightly coupled feed-forward feedback networks in the visuomotor integration along the dorsal pathway enable a graceful visually guided movement (Pratviel et al., 2021). Further knowledge of the feed-forward and feedback dynamics in the hippocampus could help design an extremely memory-efficient neuromorphic computer (Abu-Hassan et al., 2019; Lee et al., 2021).

## Data Availability Statement

The raw data supporting the conclusions of this article will be made available by the authors, without undue reservation.

## Ethics Statement

The animal study was reviewed and approved by the UC Irvine Institutional Animal Care and Use Committee.

## Author Contributions

YV and GB designed the experiments and wrote the article. GB assembled the devices and performed the network culture and recording. YV analyzed the original recordings and generated code for data processing to produce the graphics. WT and BW helped in editing and writing the applications. BW and GB secured funding for this study. All the authors contributed to interpretation and editing.

## Conflict of Interest

The authors declare that the research was conducted in the absence of any commercial or financial relationships that could be construed as a potential conflict of interest.

## Publisher’s Note

All claims expressed in this article are solely those of the authors and do not necessarily represent those of their affiliated organizations, or those of the publisher, the editors and the reviewers. Any product that may be evaluated in this article, or claim that may be made by its manufacturer, is not guaranteed or endorsed by the publisher.

## Footnotes

1
https://www.3brain.com/resources/gallery

2
www.brilliant.org/wiki/log-normal-distribution

## Supplementary Material

Supplementary Figure 1 Examples of spike sorting from axons crossing two electrodes in a single microfluidic tunnel. The goal is to pair sorted units on one tunnel electrode with the units on the other tunnel electrode. **(A)** Spike sorting on one electrode on the CA1 side of the tunnel (F9) indicates three units with spike counts. **(B)** The second electrode (G9) on the CA3 side also indicates three units. The matching algorithm examines the relative timing of the spikes to calculate normalized matching index (NMI) to match units, proceeds to assign feed-forward or feedback direction, and calculates conduction velocity (delay time/200 μm spacing of electrodes) for the matched axons. **(C)** Example of a spike delay corresponding to the FB axon with a spike delay of 0.52 ms, indicating spike propagation in feedback direction with a velocity of 0.37 m/s. **(D)** Example NMI matrix calculated between three sorted units in the CA1 side of the tunnel and three sorted units in the CA3 side of the tunnel. NMI quantifies the fraction of total spikes that are within delay times of 0.1 and 1 ms (corresponding to known conduction velocities of 0.2–2 m/s). Units with maximum NMI (above threshold of 0.2) are matched together as belonging to the same axons. Two NMI values indicated in red belong to the two axons identified, which are unit 1 in CA1 paired with unit 1 in CA3, and unit 3 in CA1 paired with unit 2 in CA3 [see also arrows in panels **(A,B)**]. The latter of the two axons has an NMI of 0.34, indicating that 34% of paired spikes are within physiological conduction time delays between the two units. **(E)** Positive conduction velocities (m/s) are interpreted as feed-forward signals, negative as feedback. **(F)** Distribution of delay times between unit 1 in CA1 side electrode and unit 1 in CA3 side electrode. This delay is centered at 0.63 ms, which corresponds to an axonal conduction velocity of 0.32 m/s, from the CA3 to the CA1 side. **(G)** Another primary axon with spikes traveling backward from CA1 to CA3, with a delay centered at −0.54 ms for a conduction velocity of −0.37 m/s.

Supplementary Figure 2 Raster plots of spontaneous spikes from the 10 MEAs used for this study. Each line is 30 s. Twelve lines at each electrode equals 5 min of recording (heavy boxes are the tunnels).

Supplementary Figure 3 Measured features of spike trains include burst properties of interspike intervals, spikes per burst, inter-burst intervals, intra-burst spike rate, and burst duration.

Supplementary Figure 4 Fractions of total spikes occurring inside bursts (*n* = 10 arrays). About 60% of spikes were inside bursts with no significant differences by analysis of variance (ANOVA). The largest difference between feed-forward and feedback axons between EC and DG did not reach significance at *p* = 0.09 [*F*(1, 15) = 3.3] (*n* = 10 arrays).

Supplementary Figure 5 Poor correlation of feed-forward axons from CA3 is not due to low activity in CA3. **(A)** Strong activity in CA3 source is poorly correlated with **(B)** corresponding time range in axons emanating from CA3 toward CA1. Ten electrodes in CA3 are shown in A. Red dots mark spikes.
