# Supplementary figures and images for: The Flow of Axonal Information Among Hippocampal Subregions: 1. Feed-Forward and Feedback Network Spatial Dynamics Underpinning Emergent Information Processing
Source: Front Neural Circuits. 2021 Aug 27;15:660837. doi: 10.3389/fncir.2021.660837 (PMC8430040; doi:10.3389/fncir.2021.660837)

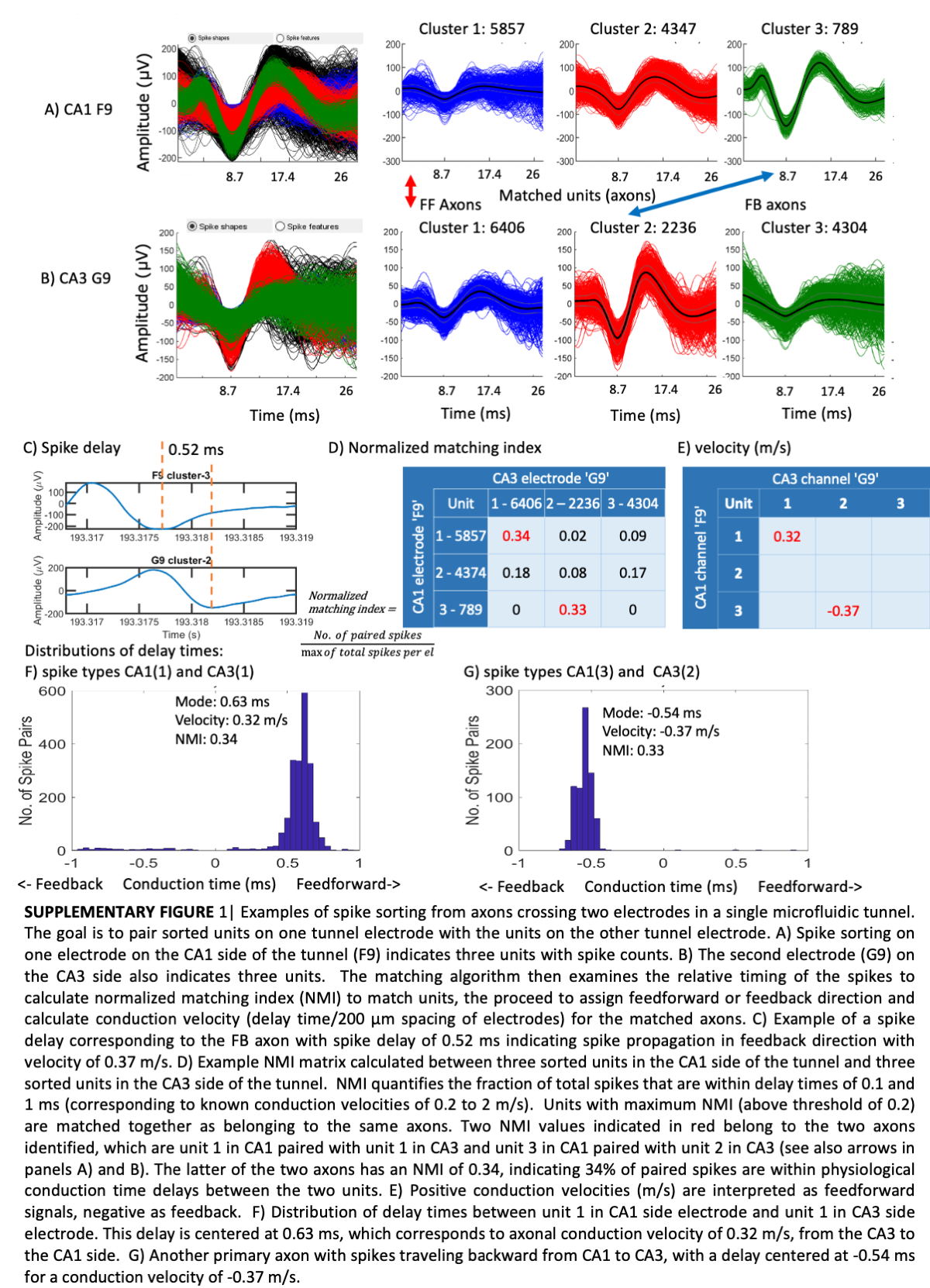

Supplement: Supplementary Figure 1 — Examples of spike sorting from axons crossing two electrodes in a single microfluidic tunnel. The goal is to pair sorted units on one tunnel electrode with the units on the other tunnel electrode. (A) Spike sorting on one electrode on the CA1 side of the tunnel (F9) indicates three units with spike counts. (B) The second electrode (G9) on the CA3 side also indicates three units. The matching algorithm examines the relative timing of the spikes to calculate normalized matching index (NMI) to match units, proceeds to assign feed-forward or feedback direction, and calculates conduction velocity (delay time/200 μm spacing of electrodes) for the matched axons. (C) Example of a spike delay corresponding to the FB axon with a spike delay of 0.52 ms, indicating spike propagation in feedback direction with a velocity of 0.37 m/s. (D) Example NMI matrix calculated between three sorted units in the CA1 side of the tunnel and three sorted units in the CA3 side of the tunnel. NMI quantifies the fraction of total spikes that are within delay times of 0.1 and 1 ms (corresponding to known conduction velocities of 0.2–2 m/s). Units with maximum NMI (above threshold of 0.2) are matched together as belonging to the same axons. Two NMI values indicated in red belong to the two axons identified, which are unit 1 in CA1 paired with unit 1 in CA3, and unit 3 in CA1 paired with unit 2 in CA3 [see also arrows in panels (A,B)]. The latter of the two axons has an NMI of 0.34, indicating that 34% of paired spikes are within physiological conduction time delays between the two units. (E) Positive conduction velocities (m/s) are interpreted as feed-forward signals, negative as feedback. (F) Distribution of delay times between unit 1 in CA1 side electrode and unit 1 in CA3 side electrode. This delay is centered at 0.63 ms, which corresponds to an axonal conduction velocity of 0.32 m/s, from the CA3 to the CA1 side. (G) Another primary axon with spikes traveling backward from CA1 to CA3, with a de [file Image_1.TIFF]

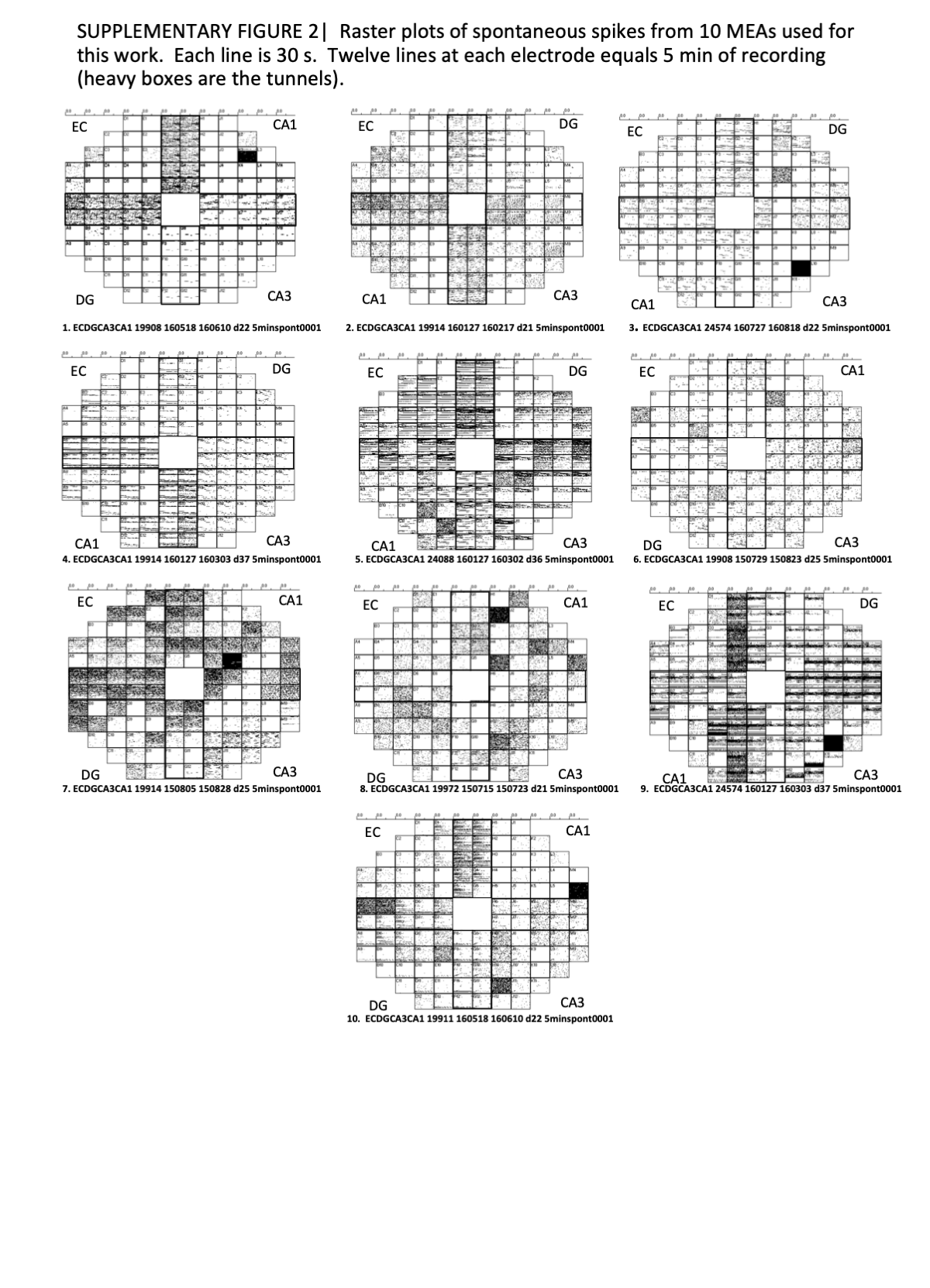

Supplement: Supplementary Figure 2 — Raster plots of spontaneous spikes from the 10 MEAs used for this study. Each line is 30 s. Twelve lines at each electrode equals 5 min of recording (heavy boxes are the tunnels). [file Image_2.TIFF]

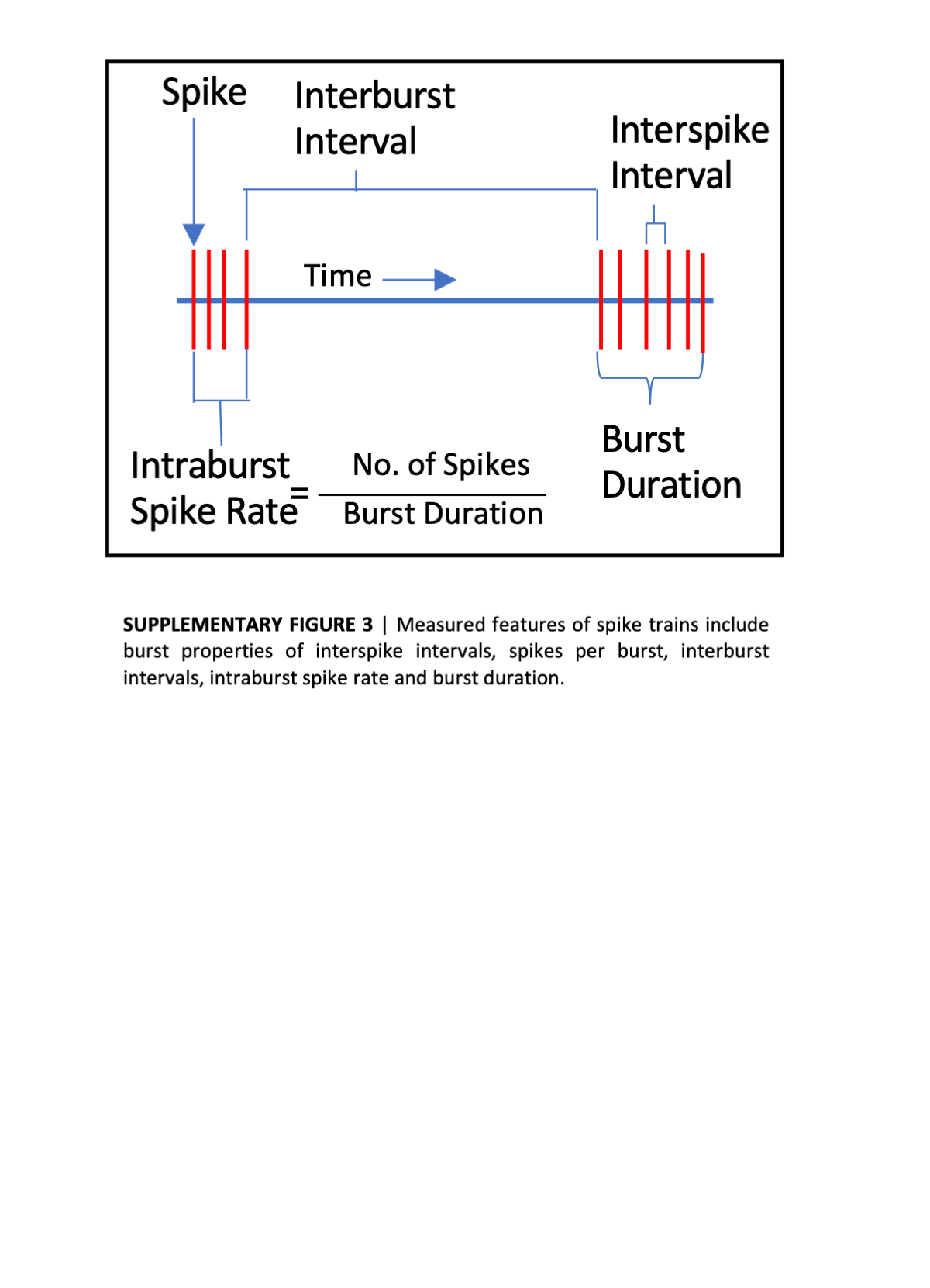

Supplement: Supplementary Figure 3 — Measured features of spike trains include burst properties of interspike intervals, spikes per burst, inter-burst intervals, intra-burst spike rate, and burst duration. [file Image_3.TIFF]

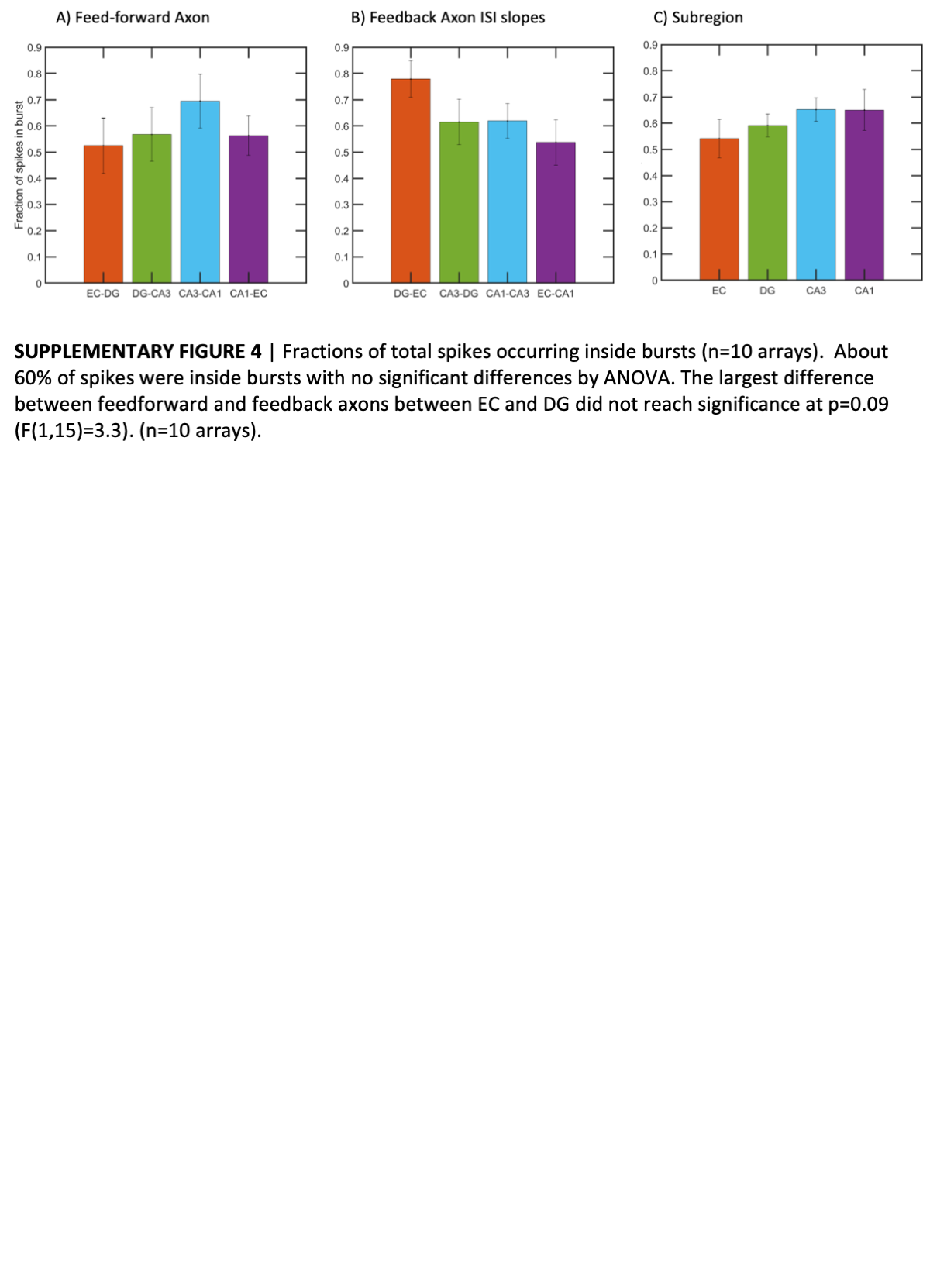

Supplement: Supplementary Figure 4 — Fractions of total spikes occurring inside bursts (n = 10 arrays). About 60% of spikes were inside bursts with no significant differences by analysis of variance (ANOVA). The largest difference between feed-forward and feedback axons between EC and DG did not reach significance at p = 0.09 [F(1, 15) = 3.3] (n = 10 arrays). [file Image_4.TIFF]

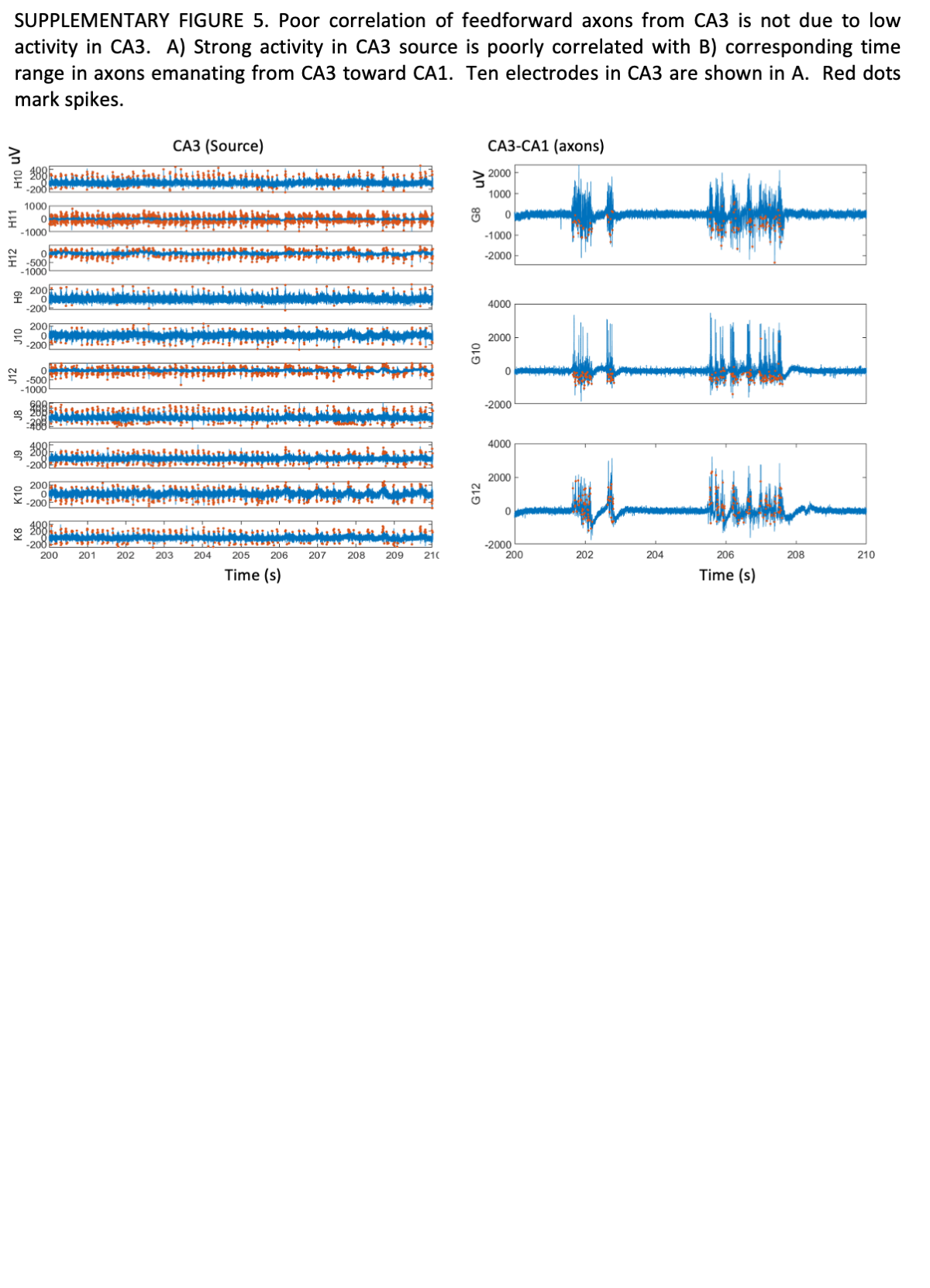

Supplement: Supplementary Figure 5 — Poor correlation of feed-forward axons from CA3 is not due to low activity in CA3. (A) Strong activity in CA3 source is poorly correlated with (B) corresponding time range in axons emanating from CA3 toward CA1. Ten electrodes in CA3 are shown in A. Red dots mark spikes. [file Image_5.TIFF]
