# Supplementary material for: The Flow of Axonal Information Among Hippocampal Subregions: 1. Feed-Forward and Feedback Network Spatial Dynamics Underpinning Emergent Information Processing
Source: Front Neural Circuits. 2021 Aug 27;15:660837. doi: 10.3389/fncir.2021.660837 (PMC8430040; doi:10.3389/fncir.2021.660837)
Supplement: Supplementary file 6 [file Table_1.docx]

SUPPLEMENTARY TABLE

**SUPPLEMENTARY FIGURE 1** | Examples of spike sorting from axons crossing two electrodes in a single microfluidic tunnel.

**SUPPLEMENTARY FIGURE 2 |** Raster plots of spontaneous spikes from 10 MEAs used for this work.

**SUPPLEMENTARY FIGURE 3** | Measured features of spike trains include burst properties of interspike intervals, spikes per burst, interburst intervals, intraburst spike rate and burst duration.

**SUPPLEMENTARY FIGURE 4** | Fractions of total spikes occurring inside bursts (n=10 arrays).

**SUPPLEMENTARY FIGURE 5** | Poor correlation of feedforward axons from CA3 is not due to low activity in CA3.

**Files used to generate results**

Home folder: \\brewerserver\shared\Yash\SCA-spike-lfp-theta\sorted-spike-analysis\exclusive-ff-vs-fb\spike-burst-dynamics\

Script names:

1. compute_wells_burst_and_spike_dynamics_200922.m
2. compute_spike_burst_dynamics_axons_200922
3. compute_tunnels_burst_and_spike_dynamics_histogram_200922
4. plot_allregion_axonal_spike_burst_dynamics_200922.mlx
5. plot_allregion_well_spike_burst__dynamics_200922.mlx
6. plot_allregion_tunnel_spike_burst_dynamics_200923.mlx

Arrays used (compartments included, array number, date of plating (YYMMDD), date of recording, days in vitro, length of spontaneous recording:

1. ECDGCA3CA1 19908 160518 160610 d22 5minspont0001
2. ECDGCA3CA1 19914 160127 160217 d21 5minspont0001
3. ECDGCA3CA1 24574 160727 160818 d22 5minspont0001
4. ECDGCA3CA1 19914 160127 160303 d37 5minspont0001
5. ECDGCA3CA1 24088 160127 160302 d36 5minspont0001
6. ECDGCA3CA1 19908 150729 150823 d25 5minspont0001
7. ECDGCA3CA1 19914 150805 150828 d25 5minspont0001
8. ECDGCA3CA1 19972 150715 150723 d21 5minspont0001
9. ECDGCA3CA1 24574 160127 160303 d37 5minspont0001
10. ECDGCA3CA1 19911 160518 160610 d22 5minspont0001
